# Usage of a simplified blumgart pancreaticojejunostomy in laparoscopic pancreaticoduodenectomy: a single center experience

**DOI:** 10.1186/s12893-023-02248-4

**Published:** 2023-11-10

**Authors:** Chuan-zhao Zhang, Zhong-Yan Zhang, Shan-zhou Huang, Bao-hua Hou

**Affiliations:** 1 Department of General Surgery, Guangdong Provincial People’s Hospital (Guangdong Academy of Medical Sciences), Southern Medical University, Guangzhou, 510080 China; 2 School of Medicine, South China University of Technology, Guangzhou, 51000 China

**Keywords:** Laparoscopic pancreaticoduodenectomy, Pancreaticojejunostomy, Pancreatic fistula, Simplified Blumgart anastomosis, Ductal to mucosa pancreaticojejunostomy

## Abstract

**Background:**

Blumgart pancreaticojejunostomy (PJ) was shown to be an effective method for pancreaticojejunostomy in open pancreaticoduodenectomy. But the original Blumgart method is involved in complicated and interrupted sutures, which may not be suitable for the laparoscopic approach. In this study, we introduced a simplified Blumgart method for laparoscopic pancreaticojejunostomy.

**Methods:**

We retrospectively reviewed 90 cases of pancreaticoduodenectomy in our institute from 2019 to 2022. Among them, 32 patients received LPD with simplified Blumgart PJ, while 29 received LPD with traditional duct-to-mucosal anastomosis (the Cattel-Warren technique) and 29 received OPD with traditional duct-to-mucosal anastomosis. And the time length for PJ and the surgical outcome were compared in these three groups.

**Results:**

The simplified Blumgart pancreaticojejunostomy was accomplished in all 32 cases with no conversion to open surgery due to improper sutures. And the time length for laparoscopic simplified Blumgart pancreaticojejunostomy was 26 ± 8.4 min, which was shorter than laparoscopic traditional ductal to mucosa pancreaticojejunostomy (39 ± 13.7 min). Importantly, the overall incidence for POPF and grade B&C POPF rate in the laparoscopic simplified Blumgart method group were 25% and 9.38% respectively, which were lower than the other two groups. Moreover, we performed univariate analysis and multivariate analysis and found soft pancreas, pancreatic ductal diameter < = 3 mm and intraoperative blood loss were independent risk factors for POPF after PD.

**Conclusion:**

Our data suggest that the simplified Blumgart method is a feasible and reliable method for laparoscopic PJ which deserves further validation.

**Supplementary Information:**

The online version contains Supplementary material available at 10.1186/s12893-023-02248-4.

## Introduction

Laparoscopic pancreaticoduodenectomy (LPD) is one of the most complex and difficult surgical procedure in abdominal surgery since it contains major resection and refined reconstruction. The corresponding complications and mortality following the operation are relatively high [1, 2]. Indeed, the incidence of postoperative pancreatic fistula (POPF) was reported to be 10–40%, which was even higher in the context of laparoscopic approach compared to open surgery [3]. Therefore, it is of great interest to find some strategies to reduce the POPF rate in LPD, including improving the surgical skills and methods.

There are many risk factors associated with POPF after receiving pancreaticoduodenectomy [4]. Among them, surgeons’ experience and the pancreaticojejunostomy methods have an important influence. The Blumgart method was introduced in 2000 [5] and it was recently reported to be associated with decreased POPF rate [6]. It is basically a trans-pancreatic U-shaped suture between the pancreatic parenchyma and the jejunal seromuscular layer, which may reduce the anastomosis tension. However, the original Blumgart method is an interrupted suture which requires 4–6 stitches in total and could be very difficult to accomplish in the laparoscopic approach. Thus, in this study, we introduce a simplified Blumgart method with only two stitches between the pancreatic parenchyma and the jejunal seromuscular layer. And we investigate the feasibility of this simplified Blumgart pancreaticojejunostomy in LPD and the patients’ outcomes receiving this procedure.

## Methods

### Study design and participants

We review the data from 90 consecutive cases of pancreaticoduodenectomy performed in the Department of General Surgery, Guangdong Provincial People’s Hospital, Guangdong Academy. Patients who met the following criteria were excluded: those with a history of pancreatic surgery; history with chronic organ insufficiency; less than 18 years old. The two primary surgeons all have 12 years of laparoscopic pancreatic surgery experience. From 2019 to 2022. 32 patients received LPD with simplified Blumgart PJ in group 1 in which double U suture were performed. 29 received OPD with traditional duct to mucosal anastomosis in group2 and 29 received LPD with traditional duct-to-mucosal anastomosis in group3. The selection for the laparoscopic simplified Blumgart method or traditional duct-to-mucosal method were decided case by case by the two responsible surgeons during the operations. Briefly, the surgeons will consider the factors including pancreas texture, pancreas duct diameter, pancreas stump thickness, bleeding tendency, etc. and they tended to perform the Cattel-Warren method if they thought it could be finished with high quality. If the Cattel-Warren method was difficult to finish they would choose Simplified Blumgart method.

### Data collection and definitions for postoperative complication

The general clinicopathological factors (age, sex, pathological diagnosis of the disease, BMI, diabetes.), preoperative factors (preoperative bilirubin, preoperative albumin, preoperative HB level ), intraoperative factors (operation time, pancreaticojejunostomy, and intraoperative blood loss, pancreatic texture, the diameter of the pancreatic duct), and postoperative results (the amylase level in serum and drainage, the total bilirubin levels in serum and drainage, hospitalization time, complications, blood transfusion ) of all patients were collected and analyzed. POPF was defined on the basis of the International Study Group of Pancreatic Fistula (ISGPF) definition [7]. The bile leakage level was defined on the basis of the International Study Group of Liver Surgery definition [8]. The postpancreatectomy hemorrhage (PPH) level was defined by the International Study Group of Pancreatic Surgery (ISGPS) definition [9]. The classification of postoperative complications followed the Clavien–Dindo classification system of 2004 [10].

### Surgical procedure of simplified blumgart pancreaticojejunostomy and traditional duct to mucosal method

In the LPD and OPD procedure, the pancreatic neck was routinely transected using a harmonic scalpel and the resection part was completed accordingly. Generally, the simplified Blumgart Pancreaticojejunostomy included a duct-to-mucosa anastomosis and U shape suturing of pancreatic parenchyma and jejunum seromuscular layer, in order to form a jejunal serosa mattress on the pancreatic stump [7].

**Fig. 1:**
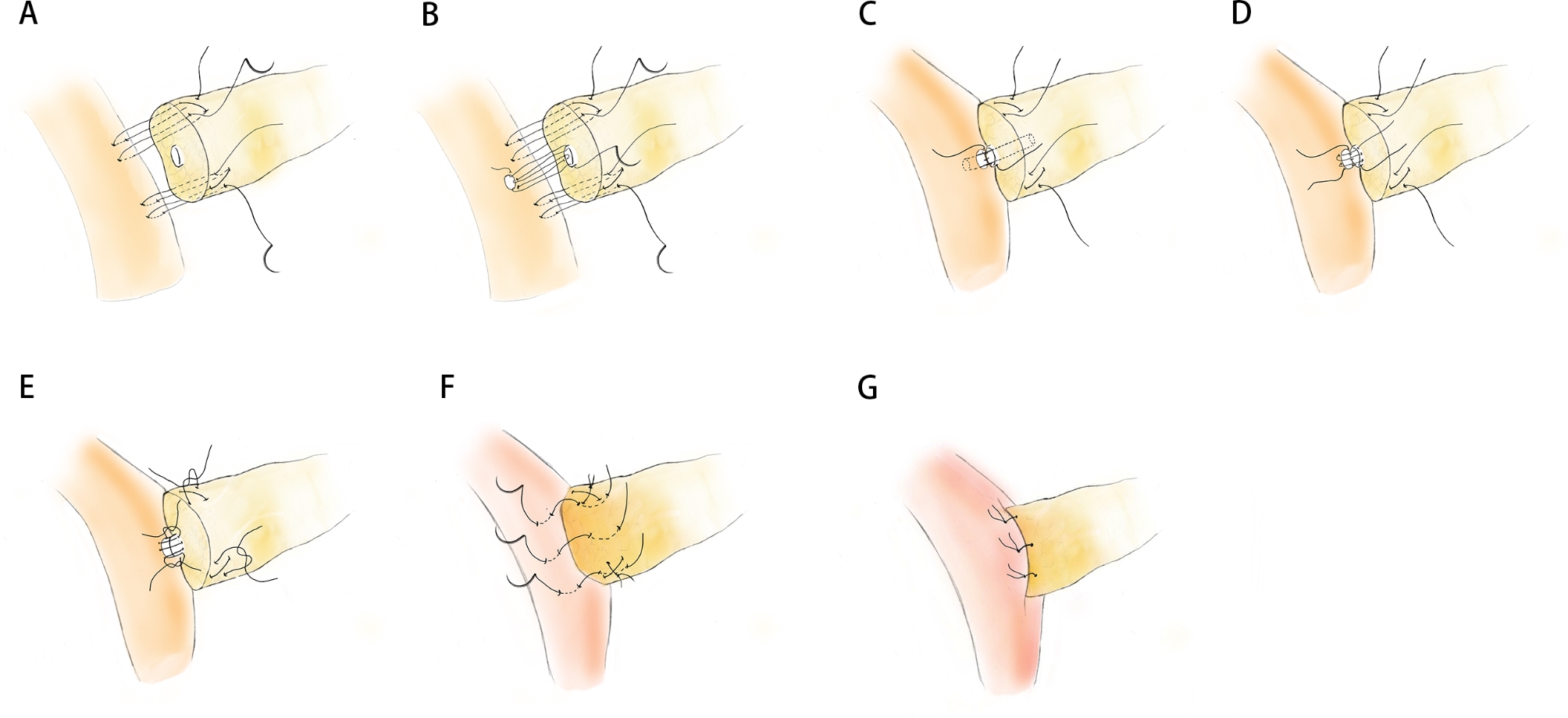
Surgical procedure of Simplified Blumgart Pancreaticojejunostomy. **A** The double U-shaped suture between the pancreatic parenchyma and the dorsal side of the jejunal seromuscular layer were performed at the superior and inferior side of the main pancreatic duct. **B** The continuous suture of the posterior semicircle of the duct-to-mucosa anastomosis. **C** The placement of pancreatic duct stent. **D** The continuous suture of the anterior semicircle of the duct-to-mucosa anastomosis. **E** Ties were made between the threads of the posterior semicircle and the anterior semicircle duct-to-mucosa anastomosis. And U shape suture were pulled under an appropriate tension and tied. **F** The interrupted suture at the jejunal ventral wall. **G** Schematic diagram when finishing the simplified PJ.

First, two U-shaped sutures were performed at the superior and inferior side of the main pancreatic duct on the pancreatic parenchyma and the dorsal side of the jejunal seromuscular layer in the direction of short axis of jejunum. The threads were clipped temporarily and not tied (Fig. 1A; Supplementary Fig. 1A, B).

The posterior semicircle of the duct-to-mucosa anastomosis was performed with 5 − 0 PDS by continuous suture (Fig. 1B; Supplementary Fig. 1C). Then a pancreatic duct stent was placed (Fig. 1C; Supplementary Fig. 1D), followed by continuous suture for the anterior semicircle of the duct-to-mucosa anastomosis (Fig. 1D; Supplementary Fig. 1E). The threads of these two continuous sutures were tied at the superior and inferior side, respectively (Fig. 1E).

The previously clipped U shape suture were pulled under an appropriate tension and tied (Fig. 1E; Supplementary Fig. 1F). Then the anterior wall of the pancreatic parenchyma and jejunum were reinforced using additional 3–4 interrupted sutures and the knots were made at the anterior wall of jejunal in case of the laceration of pancreatic parenchyma (Fig. 1F-G; Supplementary Fig. 1G, H).

The traditional duct-to-mucosal method was followed the Cattel-Warren technique [8] except the interrupted suture was replaced by continuous suture in the laparoscopic operations.

### Perioperative drain management

Two drainage tubes were placed after completion of reconstruction, with one tube placed behind the PJ area and the other tube placed at the right side of hepaticojejunostomy. Amylase level of the drainage fluid was tested daily after operation. We start removing the drainage tube on postoperative day (POD) 3 if the amylase level of drainage fluid was < 5000 U/L on POD 1 and no signs for abdominal infection on POD 3.

### Statistical analysis

SPSS 26.0 (SPSS Inc., IBM, Armonk, NY, USA) statistical software was used for data analysis in this study. Data were expressed as the mean ± standard deviation if data were conformed to normal distribution, otherwise, data were expressed as medians (25th − 75th percentiles).

One-way ANOVA, Kruskal–Wallis H test, and Mann-Whitney U test were used when comparing the three groups. Categorical variables were analyzed using ${\chi }^{2}$ test or Fisher’s exact test. All variables were incorporated into a univariate analysis. P < 0.05 was considered to be statistically significant. Statistically significant variables demonstrated in univariate analysis were incorporated in multivariate logistic regression to analyze the independent risk factors for pancreatic fistula after pancreaticojejunostomy.

## Results

### Clinical characteristics of study population

A total of 90 patients were enrolled in this study. The patients were divided into 3 groups based on the methods of pancreaticojejunostomy. There were 32 patients in group 1 who received LPD with simplified Blumgart pancreaticojejunostomy. 29 patients in group 2 received OPD with traditional ductal to mucosa anastomosis and 29 patients in group 3 received LPD with traditional ductal to mucosa anastomosis. The baseline characteristics of the enrolled patients are listed in Table 1. No significant differences were identified between the 3 groups in terms of gender, age, BMI, ASA, intraoperative blood loss, length of stay after surgery, diameter of pancreatic duct, pancreatic texture, jaundice, and diabetes.

**Table 1:** Patient characteristics and intraoperative factors

GROUP	GROUP1	GROUP2	GROUP3	F/${\varvec{\chi }}^{2}$/H	P value
Pancreaticojejunostomy					
Simplified Blumgart	32(100)	(0)	0(0)		
Traditional ductal to mucosa (Cattel-Warren technique)	0(0)	29(100)	29(100)		
Laparoscopic approach	YES	NO	YES		
N	32	29	29		
Gender(male)				${\varvec{\chi }}^{2}$=2.050	0.359
male	14	18	15		
female	18	11	14		
AGE	56.47 ± 12.31	57.52 ± 9.10	60.97 ± 11.99	F = 1.304	0.277
BMI	21.7875 ± 2.83	21.50 ± 3.20	23.25 ± 3.01	F = 2.858	0.063
Operation time (min)	303.68 ± 64.39	267.17 ± 50.45	308.01 ± 64.40	F = 4.066	0.021
Intra operative blood loss	280(100–400)	310(200–475)	250(100–450)	H = 2.883	0.237
Length of stay after surgery	16.5(13.25–28.25)	15(12.00-18.5)	19(13–24)	H = 3.215	0.200
Time length for pancreaticojejunostomy (min)	26.81 ± 3.79	23.69 ± 4.85	39.34 ± 4.21	F = 109.05	< 0.01
ASA I–II				${\varvec{\chi }}^{2}$=5.956	0.202
I	1	6	4		
II	29	23	24		
III	2	0	1		
pancreatic ductal diameter(mm)	3.11 ± 0.78	3.19 ± 0.84	2.94 ± 0.85	F = 0.700	0.499
Pancreatic texture				${\varvec{\chi }}^{2}$=1.623	0.444
Soft	17	18	20		
Hard	15	11	9		
Final pathological diagnosis					
Ampullary Adenocarcinoma	10	2	11		
Biliary cancer	5	3	0		
Pancreatic cyst tumor	5	3	4		
Pancreatic Ductal Adenocarcinoma	4	14	3		
Other diseases	8	7	11		
Jaundice(yes)				${\varvec{\chi }}^{2}$=3.057	0.217
YES	17	16	10		
NO	15	13	19		
Diabetes				${\varvec{\chi }}^{2}$=0.536	0.765
YES	4	3	2		
NO	28	26	27		

### The feasibility of simplified blumgart pancreaticojejunostomy in LPD

To study the feasibility of the simplified Blumgart pancreaticojejunostomy in laproscopic approach, we calculate the success rate of the procedure and the time length needed for the pancreaticojejunostomy. The simplified Blumgart pancreaticojejunostomy were finished in all the 32 cases with no conversion to open surgery due to improper suture or other reasons. 2 of 32 cases received a repeated procedure due to broken threads during continuous suture. And the time length for simplified Blumgart pancreaticojejunostomy were 26.81 ± 3.79 min, which was comparable to open traditional ductal to mucosa pancreaticojejunostomy (23.69 ± 4.85 min) and importantly, was shorter than laparoscopic traditional ductal to mucosa pancreaticojejunostomy (39.34 ± 4.21 min).

### Postoperative outcome and complications

We analyzed the patients’ postoperative outcomes and complications in the three groups, which were listed in Table 2. Mann-Whitney U test was applied in comparison between group 1 and group3. We found comparable incidence for PPH and Bile Leakage in the three groups. Of note, the total incidence of POPF in group 1, group 2 and group 3 were 25% (8/32), 44.82% (13/29) and 48.27% (14/29), respectively. Specifically, the rate of grade B&C POPF in the group 1, group 2, and group 3 were 9.38% (3/32), 13.79% (4/29) and 20.69% (6/29), respectively. Although there was no statistical difference when performing comparison among three groups, the comparison between group 1 and group 3 showed statistically lower total incidence and grade B&C POPF in group 1. Similar results were shown for postoperative complications based on Clavin-Dindo Classification. The total complication rate in the group 1, group 2, and group 3 were 21.87% (7/32), 44.83% (13/29) and 37.93% (11/29), respectively. And the Grade III-V complication rate were 3.1% (1/32), 10.34% (3/29), and 10.34% (3/29), respectively. These indicate patients who received simplified Blumgart pancreaticojejunostomy may have reduced incidence of clinically significant POPF and Grade III-V complications.

**Table 2:** Incidence of postoperative complications

GROUP	GROUP1	GROUP2	GROUP3	Z/t	P value (Mann-Whitney U test between GROUP1&3)	H/F	P value (Kruskal–Wallis H test within 3 groups)
Clavin-Dindo Classification				Z = 1.764	0.078	H = 4.090	0.129
Grade I	4	6	3				
Grade II	2	4	5				
Grade III-V	1	3	3				
POPF				Z = 2.081	0.037	H = 4.011	0.135
Biochemical leak	5	9	8				
Grade B	3	4	5				
Grade C	0	0	1				
Diameter of pancreatic fistula related fluid area for grade B&C POPF(cm)	2.85 ± 1.14	2.80 ± 1.38	3.19 ± 1.65	t = 0.320	0.757	F = 0.115	0.893
PPH				Z = 0.123	0.902	H = 0.478	0.788
Grade A	2	5	2				
Grade B	4	3	4				
Grade C	0	0	0				
Bile Leakage				Z = 0.367	0.713	H = 3.678	0.159
Grade A	7	2	4				
Grade B	1	0	2				
Grade C	0	0	0				

POPF: postoperative pancreatic fistula; PPH: postpancreatectomy hemorrhage

To further investigate the severity of POPF, we detected the diameter of pancreatic fistula related fluid area around surgical field by CT or ultrasound B for patients with grade B&C POPF. The results showed the diameter of the fluid area of 3 cases in group 1 was 2.85 ± 1.14 cm, which was comparable with the group 3.

### The impact of pancreaticojejunostomy methods and other risk factors on POPF

We next try to investigate the risk factors for POPF. Univariate analysis showed no significant correlation between POPF and the following factors: gender, age, BMI, diabetes, preoperative total bilirubin, serum albumin, laparoscopic approach, operative time. Of note, the parameter for pancreaticojejunostomy method did not reach a statistical difference (${\chi }^{2}$=4.030, P = 0.070). Conversely a significant correlation was observed between POPF and the following factors: intraoperative blood loss, pancreatic ductal diameter and pancreatic texture (Table 3).

**Table 3:** Risk factors for pancreatic fistula by univariate analysis

VARIABLE	PF GROUP	NPF GROUP	${\varvec{\chi }}^{2}$	P
**Sex**			0.306	0.667
**Male**	17	30		
**Female**	18	25		
**Age**			1.023	0.388
**≥ 60**	14	28		
**＜60**	21	27		
**BMI**			0.07	1.000
**≥ 25**	5	9		
**＜25**	30	46		
**Laparoscopic approach**			0.269	0.645
**Yes**	23	39		
**No**	12	16		
**Diabetes**			0.719	0.527
**Yes**	6	6		
**No**	29	49		
**Preoperative total bilirubin (µmol/L)**			0.556	0.520
**> 171**	15	28		
**≤ 171**	20	27		
**Serum albumin (g/L)**			1.336	0.283
**≥ 35**	16	32		
**＜35**	19	23		
**Pancreaticojejunostomy method**			4.030	0.070
**Simplified Blumgart**	8	24		
**Traditional ductal to mucosa**	27	31		
**Time length for pancreaticojejunostomy (min)**			0.01	1.000
**> 30**	16	25		
**≤ 30**	19	30		
**Intra operative blood loss**			5.613	0.029
**＞300**	21	19		
**≤ 300**	14	36		
**Pancreatic ductal diameter(mm)**			6.661	0.016
**＞3**	10	31		
**≤ 3**	25	24		
**Pancreatic texture**			6.194	0.015
**Soft**	27	28		
**Hard**	8	27		
**Operative time (min)**			0.01	1.000
**＞300**	13	21		
**≤ 300**	22	34		

PF: pancreatic fistula; NPF: non-pancreatic fistula

The risk factors for pancreatic fistula (intraoperative blood loss, pancreatic ductal diameter and pancreatic texture) demonstrated in univariate analysis were further incorporated into the logistic regression analysis (Table 4). The results showed that soft pancreas, intraoperative blood loss, pancreatic ductal diameter < = 3 mm were independent risk factors for pancreatic fistula after pancreaticoduodenectomy.

**Table 4:** Logistic regression for the predictors of pancreatic fistula following pancreaticoduodenectomy

VARIABLE	B	SE	Wals	P value	OR	95%CI
Pancreatic texture	1.285	0.522	6.058	0.014	3.615	1.299–10.059
Intra operative blood loss	1.153	0.491	5.512	0.019	3.167	1.210–8.289
Pancreatic ductal diameter(mm)	1.054	0.491	4.605	0.032	0.349	0.133–0.913

## Discussion

Completing pancreaticojejunostomy properly and reliably is critical and essential for laparoscopic pancreaticoduodenectomy, which not only relies on skillful technique, but also needs choosing an appropriate pancreaticojejunostomy method. In our study, we introduced a simplified Blumgart pancreaticojejunostomy in performing LPD procedure. And we found the total POPF and grade B&C POPF rate following this simplified Blumgart pancreaticojejunostomy was 25% and 9.38%, which was lower than laparoscopic traditional ductal to mucosa pancreaticojejunostomy (p = 0.037). But the comparison between the three groups was not significant due to impaired the statistical power caused by limited sample size. More data of prospective study with large case number is needed to further validate our PJ method in the future. In all, these suggest the simplified Blumgart pancreaticojejunostomy is an effective anastomosis method for LPD.

In the past decades, pancreatic surgery experts were trying to improve the methods and technique for pancreaticojejunostomy and many pancreaticojejunostomy methods were evaluated [7]. Among these methods, traditional ductal to mucosa PJ was used by most surgeons and was recommeded as one of the standard methods. The Blumgart method is also an anastomosis with ductal to mucosa suture for the pancreatic duct orifice and it emphasizes the adhesion between the pancreatic parenchyma and the jejunal seromuscular layer by performing trans-pancreatic U shape suture. Studies have shown that the Blumgart method was associated with less complications after pancreaticoduodenectomy [9]. Reduced or similar POPF rate was found in the Blumgart method compared with traditional ductal to mucosa method in OPD [10–12]. For LPD, performing the pancreaticoduodenectomy is more challenging and appropriate modification is needed. Indeed, several types of modified Blumgart methods were reported in recent years [13–15]. For example, Nagakawa et al. developed a modified Blumgart method using LAPRA-TY suture clips and found this method shortened the anastomosis time and had similar POPF rate compared with conventional group [13]. It is worth pointing out that they only make the knots once after finishing the suture and the knot points are on the jejunal seromuscular layer, which may help reduce stress on the pancreatic tissue. In our method, we make the knots after finishing the duct to mucosa anastomosis to firmly tie the dorsal side of the jejunal seromuscular layer to the pancreatic stump. The knot ligation here should be firm and help prevent pancreatic parenchyma bleeding, without generating too much tension. And the second knots are made after additional sutures to tie the ventral side of the jejunal seromuscular layer to the pancreatic stump. Indeed, we believe both methods are practical in experienced hands. We also think our method is easily to learn, not dependent on special equipment and is suitable for most cases. Combined with our findings, these results indicate Blumgart method with appropriate modification is feasible in LPD and associated with satisfactory outcome.

Along with the progress in surgical devices and surgical technique, the outcome of minimal invasive PD has been improved. However, patients’ selection and the surgeon’s experience are still important for ensuring the safety of the procedure. Similarly, patients’ condition and pancreatic local factors should be considered in choosing the pancreaticojejunostomy method. In the current study, we found soft pancreas and pancreatic ductal diameter were influential factors for pancreatic fistula after PD, which were consistent to other studies [16]. It is a critical issue to investigate whether our simplified Blumgart method could be safely used in the patients with high possibility for POPF. In our cohort, we did perform simplified Blumgart method in 18 patients with pancreatic duct ≤ 3 mm and we susccessfully accomplished the duct to mucosal anastomosis. We think the good exposure of surgical field is essential for the suture of the small pancreatic duct orifice. To ensure a good exposure, it is important not to tie the knot of the U shape suture between the pancreatic parenchyma and the jejunal seromuscular layer before finishing the duct to mucosal anastomosis. For the soft pancreas texture, the U shape transpancreatic suture of the simplified Blumgart method may help compress the capillaries of the pancreatic stump and decrease bleeding. Future studies with better design are needed to further validate the outcome of the simplified Blumgart method in patients with these risk factors. And we suggest perform open operations in cases with difficulty in exposure or cases with high bleeding tendency.

There are some limitations in this study. First, it was a retrospective study with relatively small sample size, which impaired the statistical power. Second, all the cases were from one single center, which was highly dependent on the surgeons’ experience. Prospective studies with large sample size should be designed in the institutions with advanced laparoscopic pancreatic surgery experience to validate these findings in the future.

In conclusion, our study demonstrates the simplified Blumgart method in LPD was associated with less anastomosis time length and may be correlated with decreased POPF compared with traditional ductal to mucosa pancreaticojejunostomy in LPD. These suggest the simplified Blumgart method is a feasible and effective pancreaticojejunostomy method in performing LPD in suitable patients.

### Electronic Supplementary material

Below is the link to the electronic Supplementary material.

Supplementary Material 1

Supplementary Material 2

## Data Availability

The datasets used and/or analyzed during the current study are available from the corresponding author upon reasonable request.

## Abbreviations

PJ: pancreaticojejunostomy
PD: pancreaticoduodenectomy
OPD: Open pancreaticoduodenectomy
LPD: Laparoscopic pancreaticoduodenectomy
POPF: postoperative pancreatic fistula
PPH: postpancreatectomy hemorrhage
