# Supplementary figures and images for: Usage of a simplified blumgart pancreaticojejunostomy in laparoscopic pancreaticoduodenectomy: a single center experience
Source: BMC Surg. 2023 Nov 10;23:339. doi: 10.1186/s12893-023-02248-4 (PMC10638819; doi:10.1186/s12893-023-02248-4)

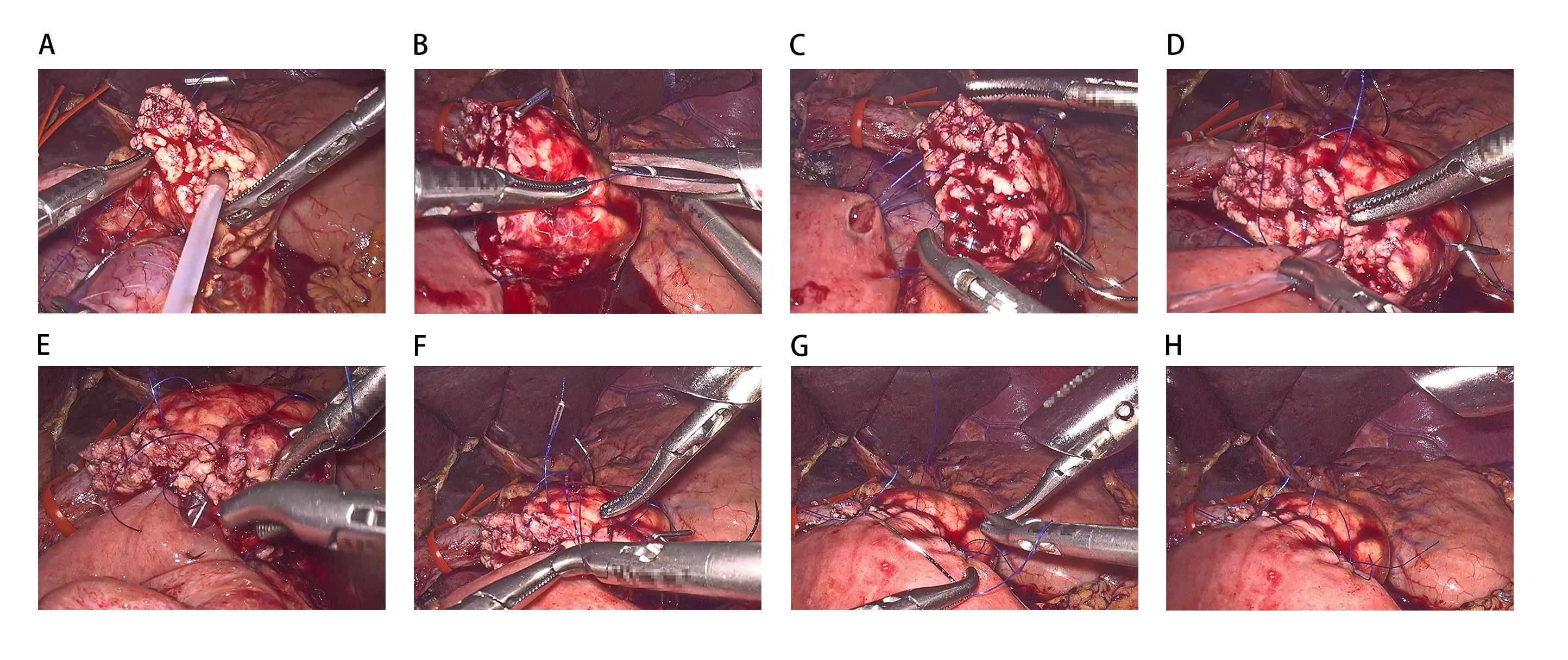

Supplement: Supplementary file 1 — Supplementary Material 1 [file 12893_2023_2248_MOESM1_ESM.tif]
