# Supplementary material for: Usage of a simplified blumgart pancreaticojejunostomy in laparoscopic pancreaticoduodenectomy: a single center experience
Source: BMC Surg. 2023 Nov 10;23:339. doi: 10.1186/s12893-023-02248-4 (PMC10638819; doi:10.1186/s12893-023-02248-4)
Supplement: Supplementary file 2 — Supplementary Material 2 [file 12893_2023_2248_MOESM2_ESM.docx]

Supplementary figure legend

Supplementary Figure1: The procedure of Simplified Blumgart Pancreaticojejunostomy. A The double U-shaped suture. **B** The temporarily fixation of the double U-shaped suture by the clip. **C** The continuous suture of the posterior semicircle of the duct-to-mucosa anastomosis. **D** The placement of pancreatic duct stent. **E** The continuous suture of the anterior semicircle of the duct-to-mucosa anastomosis. **F** Ties were made between the threads of the posterior semicircle and the anterior semicircle duct-to-mucosa anastomosis. And U shape suture were pulled under an appropriate tension and tied. **G** The interrupted suture at the jejunal ventral wall. **H** The completed anastomosis of the simplified PJ.
